# Supplementary figures and images for: Immunodetection of Truncated Forms of the α6 Subunit of the nAChR in the Brain of Spinosad Resistant Ceratitis capitata Phenotypes
Source: Insects. 2023 Nov 4;14(11):857. doi: 10.3390/insects14110857 (PMC10672392; doi:10.3390/insects14110857)

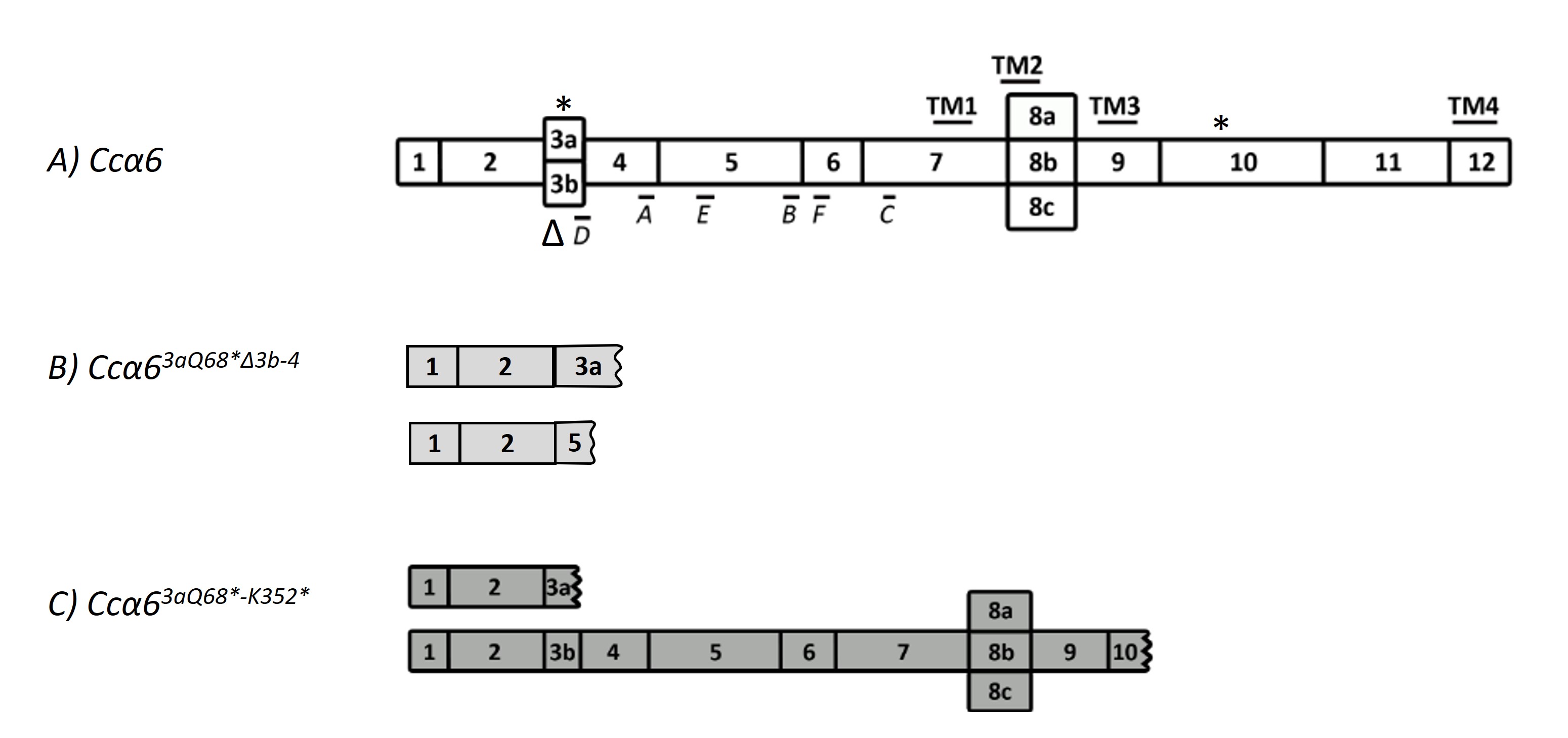

Supplement: Supplementary file 1 [file insects-14-00857-s001.zip › Figure S1.jpg]

Supplementary Figure S2

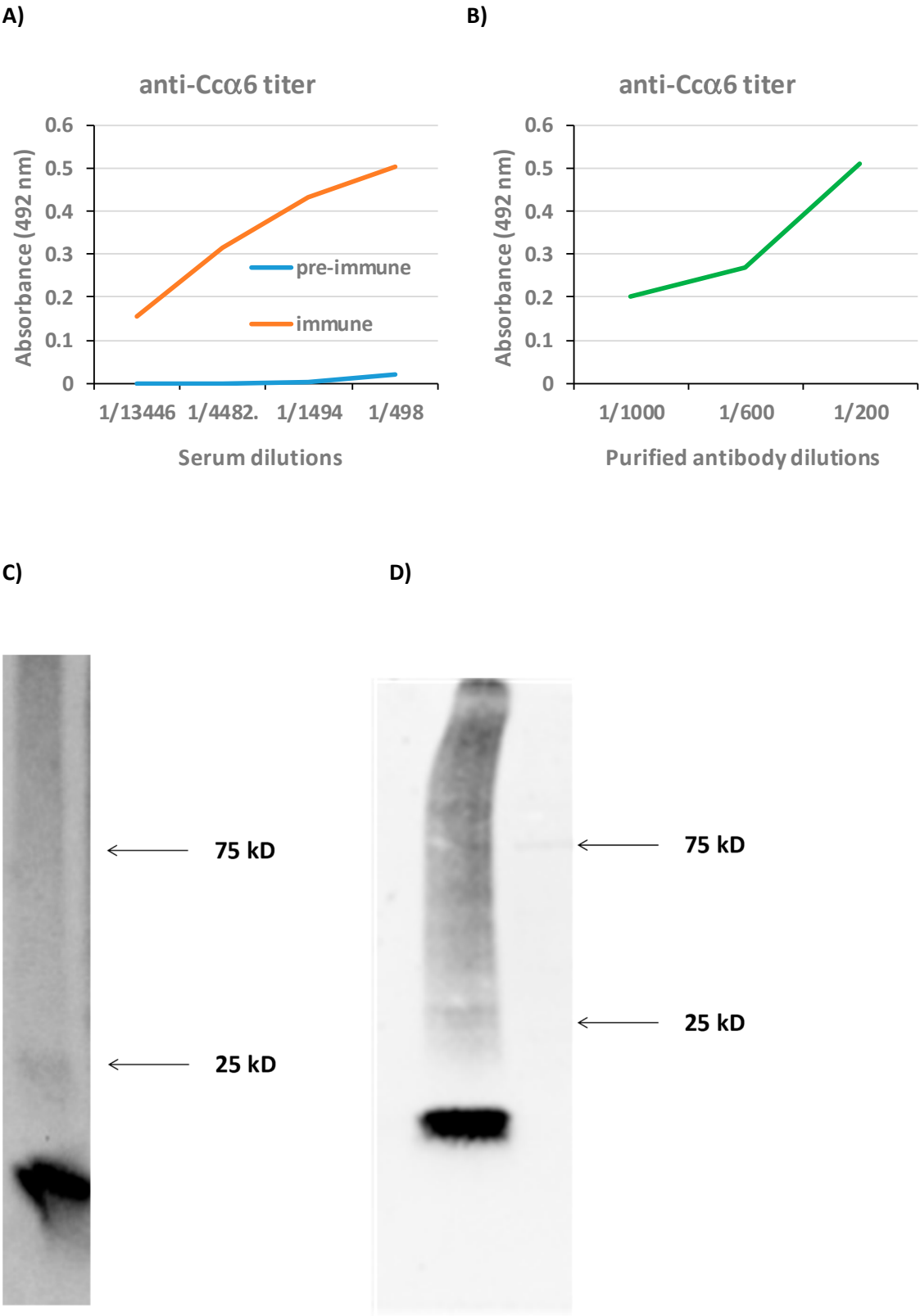

Supplement: Supplementary file 1 [file insects-14-00857-s001.zip › Figure S2.pdf]

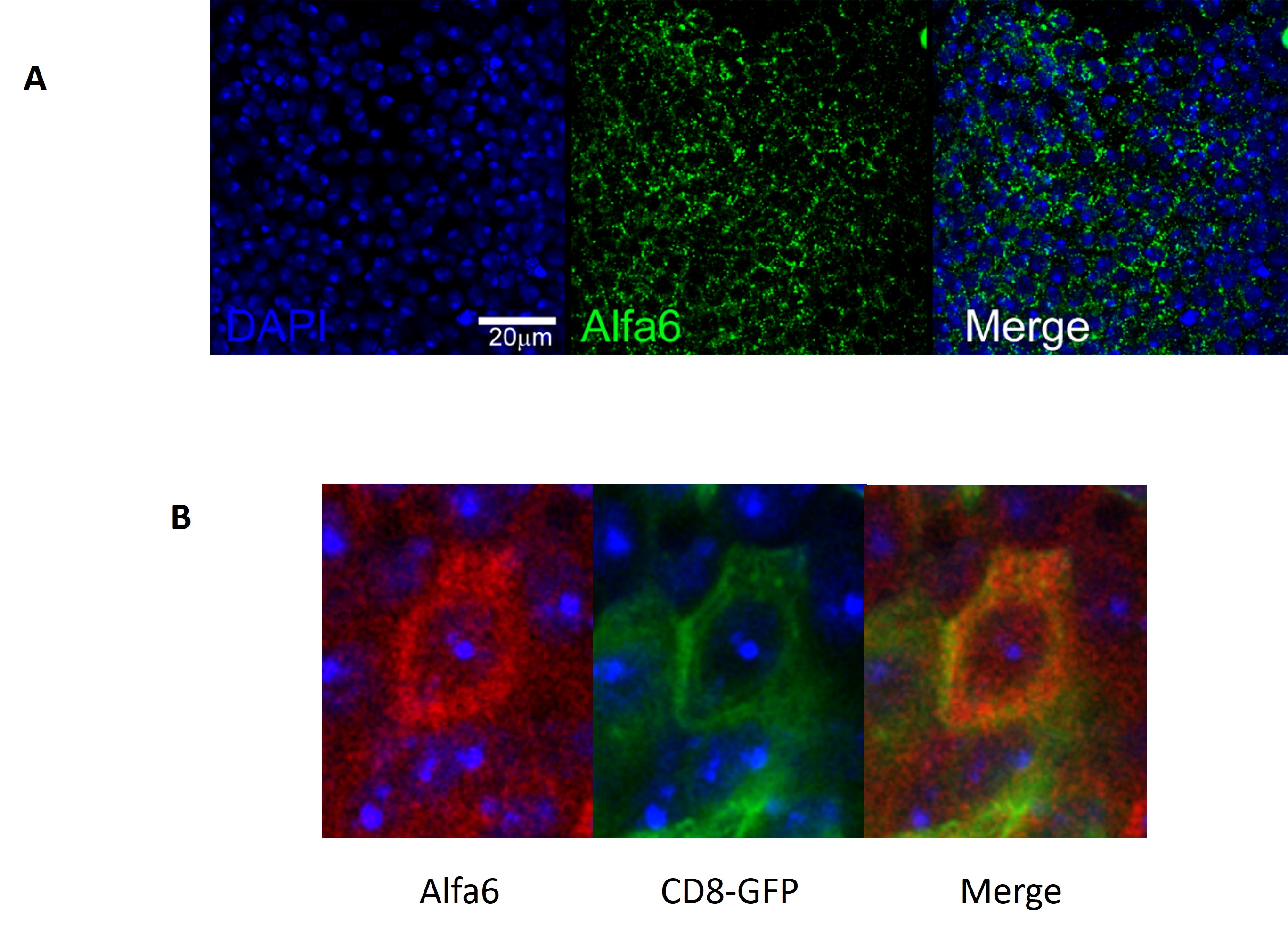

Supplement: Supplementary file 1 [file insects-14-00857-s001.zip › Figure S3.jpg]
